# Incidence and predictors of treatment interruption among patients on anti-tuberculosis treatment in Nekemte public healthcare facilities, Oromia, Western Ethiopia

**DOI:** 10.3389/fepid.2023.1234865

**Published:** 2023-12-04

**Authors:** Robsan Gudeta Getachew, Tadesse Tolossa, Zelalem Teklemariam, Angefa Ayele, Hirbo Shore Roba

**Affiliations:** ^1^ Department of Public Health, Institute of Health Sciences, Wollega University, Nekemte, Ethiopia; ^2^ School of Medical Laboratory Sciences, College Health and Medical Sciences, Haramaya University, Harar, Ethiopia; ^3^ School of Public Health, Institute of Health Sciences, Bule Hora University, Bule Hora, Ethiopia; ^4^ School of Public Health, College of Health and Medical Sciences, Haramaya University, Harar, Ethiopia; ^5^ School of Health and Medical Sciences, University of Southern Queensland, Toowoomba, QLD, Australia

**Keywords:** tuberculosis, treatment interruption, incidence, predictors, Ethiopia

## Abstract

**Introduction:**

Tuberculosis treatment interruption increases the risk of poor treatment outcomes and the occurrence of drug resistant Tuberculosis. However, data on the incidence and predictors of tuberculosis treatment interruption are still scarce in Ethiopia, as well as in the study area. Therefore, this study aimed to assess the incidence and predictors of treatment interruption among patients on tuberculosis treatment in Nekemte public healthcare facilities, Oromia region, Western Ethiopia, from July 1, 2017, to June 30, 2021.

**Methods:**

A retrospective cohort study design was conducted among 800 patients enrolled in anti-tuberculosis treatment during the study period. Data were collected from patient cards who were enrolled in treatment from July 1, 2017 to June 30, 2021. Epidata version 3.2 was used for data entry, and STATA version 14 was used for analysis. A multivariable Cox regression model with a 95% confidence interval (CI) and adjusted hazard ratio (AHR) was used to identify the significant predictors at a *p* value < 0.05. Finally, the log likelihood ratio, and a Cox-Snell residual graph was used to check the adequacy of the model.

**Results:**

A total of 800 patients were followed for a median time of 2.3 (95% CI: 2.20–2.36) months, and with a maximum follow-up time of 11.7 months. The overall incidence rate of treatment interruption was 27.4 per 1000 (95% CI: 22.8–32.8) person-month observations. Age 18–34 years (AHR = 1.8, 95% CI: 1.02–3.18), male (AHR = 1.63, 95% CI: 1.1–2.42), rural residence (AHR = 3, 95% CI: 1.98–4.64), presence of comorbidity (AHR = 10, 95% CI: 5.47–18.27) and lack of treatment supporters on the treatment follow-up (AHR = 2.82, 95% CI: 1.9–4.41) were found to be significant predictors of treatment interruption.

**Conclusion:**

A high incidence rate of interruption was observed among TB patients in public health facilities in Nekemte town. Health facilities should provide supportive care for patients with co-morbidities and consider interventions that target middle-aged patients from rural areas that reduce treatment interruptions.

## Introduction

Tuberculosis (TB) is a disease caused by *Mycobacterium tuberculosis* bacteria*,* which commonly affects the lungs (1). In Ethiopia, TB is a major public health concern, and it remains one of the highest TB burden countries, with high rates of undetected and infectious cases of TB in the population (2). One of the most important risk factors for a poor treatment outcome and the emergence of further drug-resistant TB is the interruption of treatment (3).

Approximately one-quarter of the world's population has a tuberculosis infection, and 5%–10% of infected people have a lifetime risk of contracting the disease (1). Globally, an estimated 10.6 million people became ill with tuberculosis in 2021, compared with 10.1 million in 2020, and 1.6 million people died from tuberculosis in 2021. In addition, the incidence rate of tuberculosis increased by 3.6% in 2021 relative to 2020 (4, 5). The interruption of TB treatment is characterized by missing two scheduled appointments for medication collection, whether during the intensive or continuation phases of TB treatment (6). This interruption in treatment has been associated with consequences such as delayed sputum conversion, the emergence of drug-resistant strains, extended periods of contagiousness within the community, increased mortality rates, and the need for prolonged treatment, all of which have contributed to economic and mental distress (7). The timely detection and treatment of TB are pivotal in reducing illness, fatalities, and the interruption of disease transmission (8).

Anti-TB medicines today are challenging because they are taken over a long period and because drug-resistant *Mycobacterium tuberculosis* strains are becoming more common (8). The goals of TB treatment are to cure patients as well as limit the spread of TB infection and the creation of new drug-resistant strains. These goals are not met in many parts of the world due to a variety of factors, such as severity of the illness, co-infection with HIV and/or other illnesses, poverty, and the support offered to patients, such as assisting them in taking their TB medications on time and completing their TB treatment (9).

The interruption of treatment presents a substantial obstacle in the prevention of tuberculosis and continues to pose a formidable hurdle for TB control initiatives. This is primarily because it increases the risk of severe illness, mortality, disease transmission, unfavorable treatment results, and the development of drug-resistant forms of TB (3). Similarly, studies conducted in Kenya and Kiambu showed that inadequate supervision by treatment supporters, smoking, a lack of pre-treatment counseling, distance from treatment facilities, lack of awareness of treatment length, attitudes of health workers, and TB/HIV co-infection have all been identified as major factors in TB treatment interruption (10–12).

Ethiopia has adopted and implemented strategies such as Sustainable Development Goal (SDG) number 3, which aims to improve everyone's health and wellbeing, with one of the goals being to end the global TB and HIV epidemics by 2030, and have been using the directly observed therapy short-course (DOTS) since 1991 (13). The national tuberculosis control program now has 100% geographic coverage, and DOTS is available in 92% of public hospitals and health institutions, which helps ensure the right drugs are taken at the right time for the full duration of treatment and has a paramount importance in increasing the success rate of TB treatment (14, 15). Despite the implementation of the DOTS program in Ethiopia, different reports have indicated the existence of challenges in improving TB treatment outcomes. The challenges are emanated from differences in treatment-seeking behavior, poor compliance, presence of co-infection, variations in experts’ qualification, and the presence of drug-resistant TB. Similarly, progress toward ending the epidemic and eventually eliminating tuberculosis is also slow due to limited access to health services, insufficient health infrastructure, insufficient quality of care, insufficient human and financial resources for health, and insufficient social protection (16).

Several efforts have been launched in Ethiopia to improve treatment adherence and treatment success among patients with tuberculosis. Ethiopia has made substantial progress in the detection and successful treatment of TB cases. However, the country continues to grapple with various challenges in its efforts to combat TB, and one persistent issue is the number of patients who discontinue their TB treatments. A study conducted in Western Ethiopia showed that the prevalence of TB retreatment cases was 12.2% (17) and poor treatment outcome was 30% (18). However, data on the incidence and predictors of treatment interruption are still scarce in Ethiopia, as well as in the study area. Therefore, the aim of the present study was to assess the incidence and predictors of treatment interruption among patients placed under DOTS TB treatment in Western Ethiopia.

## Methods

### Study setting and study period

This study was conducted in public healthcare facilities of Nekemte town, Oromia region, which is located 331 km from Addis Ababa. There are four public healthcare facilities in Nekemte town: one teaching referral hospital; one specialized hospital; and two health centers. In total, 951 patients with TB were enrolled in DOTS and started TB treatment follow-up in Nekemte public healthcare facilities between 1 July 2017 and 30 June 2021, and data were retrieved between 1 November and 30 November 2021.

### Study design

A retrospective cohort study design was used.

#### Study population

The study included all patients with TB who received DOTS TB treatment at Nekemte public healthcare facilities between 1 July 2017 and 30 June 2021 and whose medical records were accessible. Patients who transferred in from another health facility without full information or whose treatment outcomes were not reported in the TB registration logbook were excluded from the study.

#### Sampling procedure

All public health facilities in Nekemte town were included in the study. Initially, a list of patients with TB was retrieved from patient medical cards and the TB registration logbook. From a total of 951 patients with TB, 32 patient cards were not available, 45 cards were excluded due to the patient being under 18 years of age, and 74 cards were excluded due to incomplete information (date of initiation of TB treatment, continuation phase, and outcome not recorded). Finally, 800 patient cards with complete data were included in the final analysis, and the profiles of all patients enrolled in the TB treatment follow-up between 1 July 2017 and 30 June 2021 were reviewed.

#### Data collection instruments and procedures

The data for this study were collected using a checklist that was developed by reviewing existing literature and studies, as well as by referencing TB registration logbooks, patient medical cards, and laboratory requests. The data collection instruments contained three sections: demographic, clinical characteristics, and laboratory-related factors.

#### Variables

The dependent variable of the study was the time to the interruption of treatment. Interruption to tuberculosis treatment was defined as patients who failed to attend two scheduled appointments to collect drugs either in the intensive or continuation phases of tuberculosis treatment (4). The survival time was the time in a month from the beginning of anti-TB treatment to the development of the treatment interruption. If the patient did not develop an outcome (cured, completed, transferred out, moved to drug resistance tuberculosis (DR-TB) clinic, lost to follow-up (LTFU), died, received treatment when the study completed, and treatment failure), they were categorized under censored data. They were categorized as cured if the patients had finished treatment with a negative bacteriological result at the end of the treatment. Treatment was completed when the patients had finished treatment but lacked bacteriological results at the end of the treatment. LTFU is for patients who did not start treatment or whose treatment was interrupted for 2 consecutive months or more. Patients whose sputum smear or culture was positive at 5 months or after during treatment were considered to have treatment failure.

Independent variables include demographic variables such as age, sex, residence, population category, and linkage to TB service. Clinical aspects include the presence of comorbidities, prior TB treatment, types of TB, HIV co-infection, DOT support, nutritional status, and laboratory-related variables, such as sputum result, X-rays, and Gene Xpert result.

### Data quality control

To ensure the quality of data, the availability of all variables was checked before data collection from the TB registration book. A pretest was conducted in a Nekemte specialist hospital on 5% of patient records before the actual data collection time to assess the quality of the questions, terms, and time required to complete the checklist. Data were collected by trained health professionals. One supervisor was recruited to supervise the overall activity of data collection. One-day training was given for data collectors and the supervisor by the principal investigator. During the data collection period, the checklist was checked for completeness and consistency every day by the principal investigator.

### Data processing and statistical analysis

Data were manually checked for completeness and cleaned before being entered into Epidata version 3.2 and exported to STATA version 14 for analysis. Before analysis, simple frequencies and cross-tabulation were performed, as well as the recategorization and categorization of categorical and continuous variables. The probability of survival was estimated using a descriptive non-parametric survival analysis. Months were used as a time scale to calculate the time until the TB treatment was interrupted. To estimate survival curves for different variable categories, a non-parametric estimator, such as the Kaplan‒Meier estimator, was used. The log-rank test was used to determine significant differences between groups (19). The overall survival function and separate estimates for the stratum of covariates were considered statistically significant at a *p*-value of 0.05 in the log-rank test. A Cox proportional hazards regression model was used to determine the predictors of treatment interruption by controlling for confounding factors. Factors that were associated with outcome variables at a significance level of 25% (*p* < 0.25) in the bivariable test were included in the final multivariable analysis. The hazard ratio (HR) with 95% confidence intervals (CI) was computed, and statistical significance was declared when it was significant at the 5% level (*p* < 0.05). The proportionality hazard assumption was tested graphically (log-log plot) and Schoenfeld residuals were used to test the proportionality hazard assumption for both continuous and categorical covariates. A Cox–Snell residual plot was used to assess the overall goodness of fit of the proportional hazard model.

### Ethical considerations

Ethical approval for the study was obtained from the Haramaya University College of Health and Medical Science Institutional Health Research Ethics Review Committee (IHRERC). Since the de-identified data were extracted from patient medical cards, informed consent was not applicable to this study. Neither the case records nor the data extracted were used for any other purpose.

## Results

### Sociodemographic characteristics

Among the 800 patients observed, the mean ± standard deviation (SD) age of the participant was 32.6 ± 13.25 years. The majority (63.8%) of the patients were in the 18–34-year age group, followed by the 35–44-year age group (17.4%). More than half (60%) of the patients were male, and two-thirds (67.4%) were urban residents. In total, 607 (75.9%) patients were linked to TB services from public health facilities, and 132 (16.5%) were from PPM sites for the diagnosis of TB or to initiate anti-TB treatment (Table 1).

**Table 1:** Sociodemographic characteristics of TB patients in public healthcare facilities in Nekemte town (*N* = 800).

Variable	Category	Number	Percent (%)
Age	18–34	510	63.8
	35–44	139	17.4
	45–54	77	9.6
	≥55	74	9.3
Sex	Male	480	60
	Female	320	40
Residence	Urban	539	67.4
	Rural	261	32.6
Population category	General population	594	74.2
	Other*	206	25.8
Linkage to TB service/referred from	PHF	607	75.9
	HP	61	7.6
	PPM	132	16.5

PHF, public health facility; HP, health post; PPM, private public mix.

* Others: University students, project workers, and congregated workers.

### Clinical and laboratory characteristics

Of the 800 patients, 731 (91.5%) were newly diagnosed patients. In addition, 663 (82.9%) of the patients had a documented history of contact with individuals who had TB. Among the participants, 83 (10.4%) individuals were identified as being at risk of developing TB. A total of 56 (67.5%) were co-infected with HIV, and 17 (20.5%) were partners of people living with HIV (PLHIV). Regarding baseline methods of diagnosis, 405 (50.6%) patients were diagnosed by both acid-fast bacilli (AFB) and Gene Xpert, followed by X-ray in 189 (23.6%) patients. A total of 649 (68.6%) participants had pulmonary tuberculosis, and 251 (31.4%) had extrapulmonary TB (EPTB). The most common EPTB was TB lymphadenitis in 92 (36.6%) patients and bone TB (30%) (Table 2).

**Table 2:** Clinical and laboratory characteristics of TB patients in public healthcare facilities of Nekemte town (*N *= 800).

Variable	Category	Number	Percent (%)
Patient category	New	731	91.4
	Relapse	19	2.4
	Transfer in	50	6.3
History of contact to TB patients	Yes	663	82.9
	No	137	17.1
TB at most risk group	Yes	83	10.4
	No	717	89.6
Types of risk group	FCSW	3	3.6
	partners of PLHIV	17	20.5
	HIV co-infected	56	67.5
	prisoners	7	8.4
Diagnosis methods	AFB microscopy	129	16
	GeneXpert	26	3.3
	x-ray	189	23.6
	AFB + GeneXpert	405	50.6
	FNA/U/S	51	6.5
Types of TB	Pulmonary	549	68.7
	Extrapulmonary	251	31.4
Types of EPTB	Miliary TB	17	6.8
	Bone TB	83	33
	TB pleural effusion	59	23.5
	TB lymphadenitis	92	36.7
Nutritional status	MAM (BMI < 16)	51	6.4
	SAM (BMI 16–17.5)	61	7.6
	Normal (BMI > 17.5)	688	86
HIV test result	Negative	723	90.4
	Positive	77	9.6
DOTs supporter on the follow-up	Yes	714	89
	No	86	11

FCSW, female commercial sex workers; PLHIV, partners of people living with HIV; FNA, fine needle aspiration; U/S, ultrasound; AFB, acid-fast bacilli; MAM, moderate acute malnutrition; SAM, severe acute malnutrition; DOTs, directly observed therapy short course.

### Treatment outcomes of patients with patients

In summary, 118 (14.8%) patients experienced an interruption during their TB treatment, while the majority (*n*=682, 85.2%) did not interrupt their treatment. Of them, 331 (41.4%) patients were cured, 287 (36%) successfully finished the TB treatment program, 10 (1.2%) were transferred to the DR-TB follow-up clinic, 22 (2.3%) were moved to other medical facilities before finishing the program, 23 (2.8%) died, and nine (1%) received LTFU.

### Kaplan–Meier survival function

**Figure 1:**
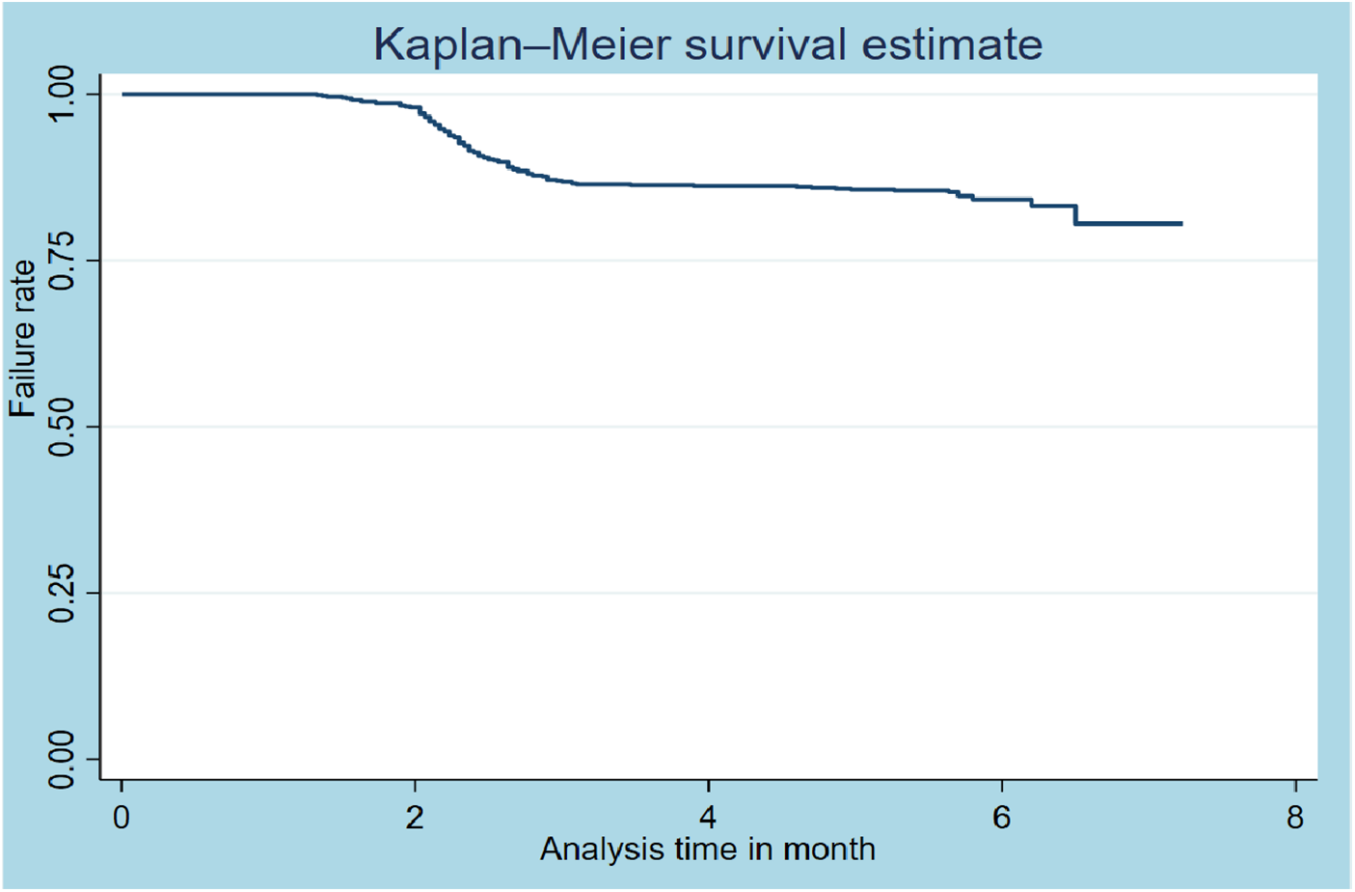
Kaplan–Meier survival function estimates of TB patients (*N* = 800).

**Figure 2:**
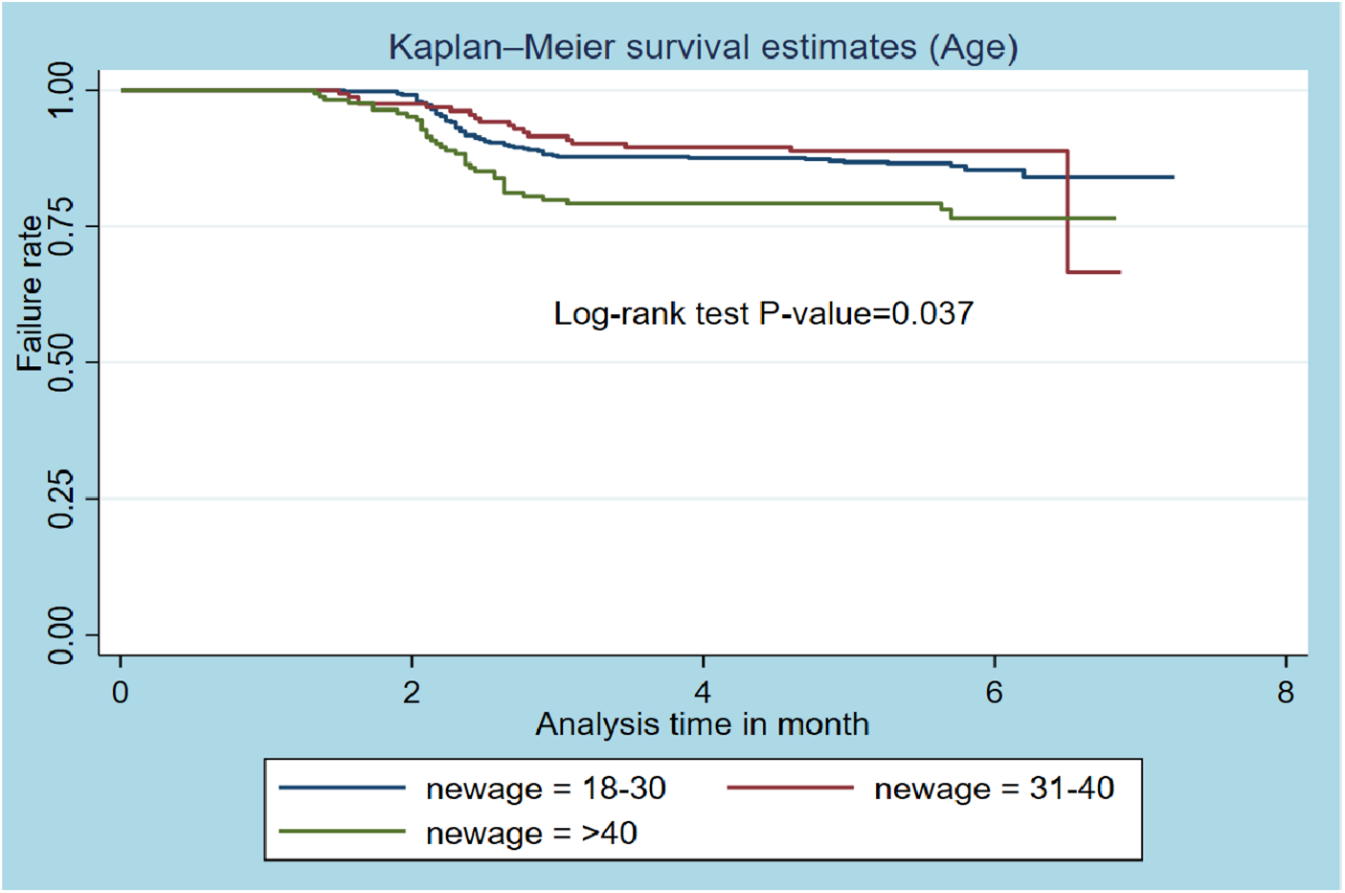
Kaplan–Meier survival estimates based on age of the patient (*N *= 800).

The overall graph of Kaplan‒Meier survivor function shows a slow decrement of events over a follow-up period (Figure 1). The Kaplan‒Meier plot for the age of the participants shows that the survival probability of patients with TB aged 35–44 years was higher than that of older (≥55 years) patients. The log-rank test revealed a statistically significant difference in survival probability across patient age groups (*p*-value = 0.0367) (Figure 2).

**Figure 3:**
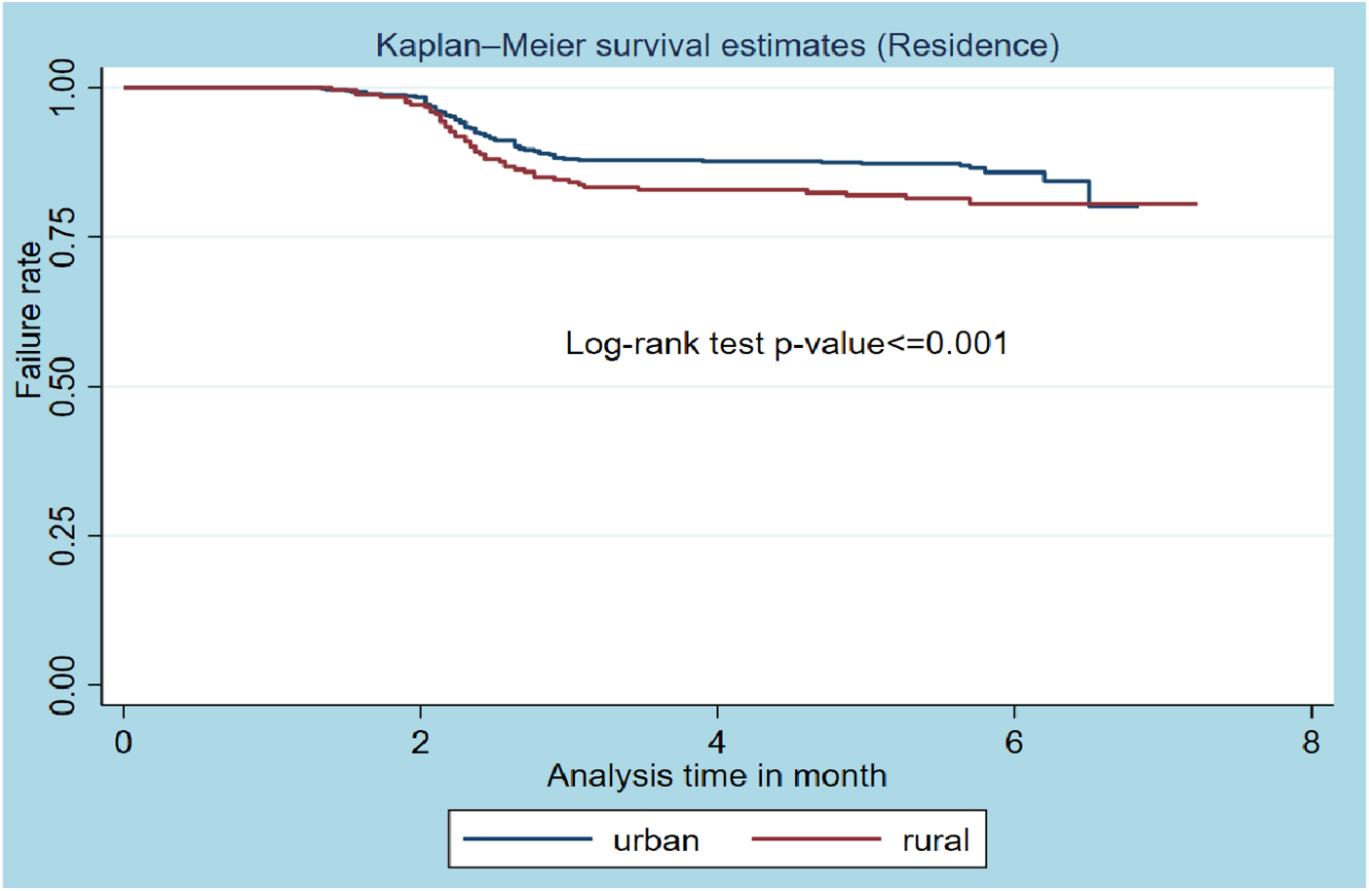
Kaplan–Meier survival estimates based on the residence of the patient (*N* = 800).

From the Kaplan‒Meier graph of residence, urban residents were more likely to survive than patients who lived in rural areas, and the log-rank test showed that there was a significant difference in the survival probability of urban and rural residents (*p*-value = 0.0000) (Figure 3).

### Incidence rate of the interruption of TB treatment

During follow-up, a total time risk of 4,304.133 person-months (PM) was observed with a minimum follow-up time of 1.2 months and a maximum follow-up time of 11.7 months. Overall, 118 (14.8%) patients with TB interrupted their TB treatment, with an incidence rate of 27.4 per 1,000 person-months (95% CI: 22.8–32.8) of observations. Most of the patients interrupted treatment in the first 3 months of follow-up, with an incidence rate of 47.54 per 1,000 PM. The incidence rate of the interruption of TB treatment was 25.6/1,000 PM observations for participants aged 18–34 years. The incidence rate of rural residents was 64.45 per 1,000 PM observations, and the interruption rate among participants linked to TB from public health facilities was 27.98/1,000 PM. Moreover, the highest incidence of interruption of TB treatment was reported in 2019 (37.63/1,000 PM) followed by 2020 (37.03/1,000 PM) (Table 3).

**Table 3:** Incidence rate of TB treatment interruption based on sociodemographic characteristics of patients in public healthcare facilities of Nekemte town (*N *= 800).

Variable	Category	Person-months	Treatment interruption	Incidence rate/1,000 PMO	95% CI
Age	18–34	2,807.56	72	25.6	20.36–32.30
	35–44	750.83	16	21.3	13.05–34.8
	45–54	382.13	11	28.78	15.94–51.9
	≥55	363.6	19	52.25	33.33–81.92
Sex	Male	2,562.26	65	25.37	19.89–32.35
	Female	1,741.86	53	30.43	23.25–39.83
Residence	Urban	3,047.4	37	12.14	8.79–16.75
	Rural	1,256.76	81	64.45	51.83–80.13
Population category	General population	3,178.4	86	27	21.90–33.42
	Others*	1,125.7	32	28.42	20.10–40.19
Linkage to TB service/referred from	PHF	3,287.67	92	27.98	22.81–34.32
	HP	311.93	8	25.64	12.83–51.28
	PPM	704.53	18	25.55	16.0–40.55
Year	2017	987.46	31	31.39	17.92–38.67
	2018	425.06	9	21.17	9.41–37.63
	2019	770.56	29	37.63	24.02–51.09
	2020	1,052.96	39	37.03	25.45–48.49
	2021	776.86	9	11.58	6.02–22.26

PMO, person-month observation; CI, confidence interval.

* Others: University students, project workers and congregated setting workers.

### Incidence rate of the interruption of TB treatment based on clinical and laboratory characteristics

In terms of clinical and laboratory characteristics, the incidence rate of treatment interruption among new patients was 24.46/1,000 PM. Patients diagnosed with Gene Xpert had an incidence rate of 44.99 per 1,000 PM, while extrapulmonary TB had an incidence rate of 30.12/1,000 PM (Table 4).

**Table 4:** Incidence rate of TB treatment interruption based on clinical and laboratory characteristics of patients in public healthcare facilities of Nekemte town (*N *= 800).

Variable	Category	Person-months	Treatment interruption	Incidence rate/1,000 PMO	95% CI
Patient category	New	3,965.23	97	24.46	20.0–29.85
	Relapse	69.8	11	157.59	87.27–284.56
	Transfer in	269.1	10	37.16	20–69
History of contact to TB patients	Yes	3,584.9	98	27.3	22.43–33.32
	No	719.23	20	27.8	17.94–43.10
TB at most risk group	Yes	461.93	8	17.3	8.66–34.63
	No	3,842.2	110	28.63	23.75–34.5
Diagnosis methods	AFB	657.16	12	18.26	10.37–32.15
	Gene Xpert	111.13	5	44.99	18.73–108
	x-ray	1,164.9	37	31.76	23–43.83
	AFB + GeneXpert	2,043.56	58	28.38	21.9–36.7
	FNA/U/S	327.33	6	18.33	8.23–40.8
Types of TB	Pulmonary	2,743.7	71	25.87	20.50–32.65
	Extrapulmonary	1,560.4	47	30.12	22.63–40
Nutritional status	MAM (BMI < 16)	267.16	8	29.9	14.97–59.87
	SAM (BMI 16–17.5)	319.5	8	25	12.5–50
	Normal (BMI > 17.5)	3,717.43	102	27.43	22.59–33.3
HIV test result	Negative	3,868	110	28.44	23.59–34.28
	Positive	436	8	18.34	9.17–36.68
DOTs supporter on follow - up	Yes	3,976	63	15.8	12.4–20.3
	No	328	55	167.6	128.7–218

CI, confidence interval; PMO, person-month observation.

### Predictors of interruption of TB treatment

Age, sex, residence, TB at-risk group (HIV co-infected, prisoners, partners of PLHIV, and female commercial sex workers), patient category, diagnosis methods, types of TB, history previous treatment, comorbidity, history of hospitalization, patients started continuation phase, and treatment supporter were candidates for multivariable Cox regression (Table 5).

**Table 5:** Predictors of treatment interruption among patients on TB treatment in public healthcare facilities of Nekemte town, from 1 July 2017 to 30 June 2021 (*N *= 800).

Variables	Category	Treatment outcome		CHR	AHR
		Interrupted *N* (%)	Uninterrupted *N* (%)		
Age	18–34	72 (14.1)	438 (85.8)	0.51 (0.31–0.85)	1.8 (1.02–3.18)*
	35–44	16 (13.6)	123 (18)	0.42 (0.21–0.82)	1 (0.5–2.0),0.98
	45–54	11 (9.32)	66 (9.7)	0.57 (0.27–1.2)	0.66 (0.31–0.14)
	>55	19 (16.1)	55 (8)	1	1
Sex	Male	65 (13.54)	415 (86.45)	1.23 (0.85–1.77)	1.63 (1.10–2.42)*
	Female	53 (16.5)	267 (83.4)	1	1
Residence	Urban	37 (6.86)	502 (93.13)	1	1
	Rural	81 (31)	180 (68.96)	5.18 (3.5–7.6)	3 (1.98–4.64)**
History of Previous TB treatment	Yes	33 (64.7)	18 (35.3)	4.5 (2.61–7.76)	1.09 (0.65–1.80)
	No	185 (24.7)	664 (88.65)	1	1
Comorbidity	Yes	80 (51.6)	75 (48.4)	1.64 (1.06–2.51)	10 (5.47–18.27)**
	No	38 (5.89)	607 (94.1)	1	1
TB treatment supporter	Yes	63 (8.8)	651 (91.2)	1	1
	No	55 (64)	31 (36)	5.9 (3.96–8.88)	2.82 (1.80–4.41)**
History of hospitalization	Yes	60 (45.8)	71 (54.2)	1.59 (0.95–2.66)	1.02 (0.60–1.73)
	No	58 (8.7)	611 (91.3)	1	1
Types of TB	Pulmonary	71 (12.9)	478 (87)	1	1
	Extrapulmonary	47 (18.7)	204 (82.8)	1.38 (0.95–2.01)	0.99 (0.67–1.46)

* *p-*value <0.05.

** *p-*value <0.001.

In the present study, the age of the patients was significantly associated with interruption of TB treatment. The 18–34-year age group had 1.8 (AHR = 1.8, 95% CI: 1.02–3.18) times higher risk of developing treatment interruption than older patients. Similarly, male patients increased the risk of interruption of TB treatment by 1.63 (AHR = 1.63, 95% CI: 1.1–2.42) than female patients.

The risk of interruption of TB treatment was three times (AHR = 3, 95% CI: 1.98–4.64) higher among participants living in rural areas than among those living in urban areas. Similarly, the risk of interruption of TB treatment was 10 times higher in patients with a history of comorbidity than in those without a history of comorbidity (AHR = 10, 95% CI: 5.47–18.27). The absence of treatment support significantly associated with TB treatment interruption. Patients lacking a treatment supporter had a 2.82 times higher risk (AHR = 2.82, 95% CI: 1.9–4.41) compared to those who had support (Table 5).

## Discussion

This study revealed that the cumulative incidence of treatment interruption was 27.4% (95% CI: 22.8–32.8). This finding is in line with a cohort study conducted in high TB burden countries such as Ethiopia (5%–25.2%) (20) and Malaysia (23.7%) (7).

On the other hand, the cumulative incidence of treatment interruption at the end of the study was 8.5% in Kenya (8), 6.5% for new patients with Smear-positive pulmonary TB in Nairobi, Kenya (21), 20% in Cameroon (22), and 7.4% in south India (23), which is lower than the current study. This variation might be due to the exclusion of patients who had been receiving treatment for longer than 3 weeks (21), but the discontinuation of treatment occurred frequently during the intensive phase (the first 2 months) of treatment in this study.

The occurrence of isoniazid (INH) treatment interruption showed variations among different ethnic groups: it was 9.7% for Asian patients, notably higher at 20.5% among white American patients, 16.1% among black patients, and 15.4% for other ethnic groups (24). Furthermore, it is noteworthy that the incidence rate in Florida is lower compared to the findings reported in this study. The difference could be because the study only included a 9-month isoniazid TB treatment course, with no other TB treatments included.

Interruption of treatment was higher in this study during the intensive phase of treatment follow-up compared to the continuation phase of treatment. These results are consistent with studies conducted in health facilities in southern Ethiopia (25), Kiambu County (10), the state of Malaysia (7), Kenya (8), Nairobi County (26), Kuwait (27), and southern India (23), in which the majority of patients interrupted treatment during the intensive phase of treatment. This might be due to differences in the age of participants and sociodemographic characteristics of participants.

Age had a statistically significant association with the interruption of TB treatment, with patients aged 18–34 years having a higher risk of treatment interruption than those aged 55 years and older. This is in line with studies conducted in Afar, Ethiopia (28), and South Africa (29). Patients aged 15–40 years and 25–34 years had a higher chance of interrupting their TB treatment. The findings of the present study differ from study reports from Kenya (8) and Russia (30); the 37–47-year and 25–50-year age groups had a higher risk of interrupting TB treatment. The differences in predictors between these studies could be due to differences in sample size and socioeconomic status in the study populations.

Another significant factor observed to influence the interruption of treatment was being male. Male patients had a higher risk of interruption of TB treatment than female patients. This finding is consistent with studies conducted in eastern Ethiopia (28), South Africa (29), Kuwait (27), Kenya (8), and Russia (30). This could be explained by the fact that employed male patients face work-related issues that affect treatment adherence, such as obtaining sick leave for treatment and fear of losing their jobs or being fired.

In this study, residing in rural areas showed a statistically significant association with the interruption of TB treatment. This finding is supported by studies conducted in TB hospitals in Jima (31), Arsi zone (32), Ghana (33), and Russia (30). In addition, a study carried out in Pakistan (31) showed a significantly increased risk of treatment interruption among those who need to travel to get medicine. This could be explained by the fact that in developing countries, such as Ethiopia, patients in rural areas have limited access to treatment and health facilities that provide anti-TB treatment.

In this study, comorbidity and lack of treatment support during treatment follow-up were also significantly associated with the interruption of TB treatment. These findings are in line with studies conducted in hospitals in Kuwait (27) and Russia (30) and a systematic review conducted in countries with a high TB burden, which shows that patients with comorbidities had a higher probability of interrupting TB (20). This study finding is in contrast with the study carried out in Florida (24), which found that the risk of treatment interruption was lower among patients with diabetes and those with one or more immunosuppressive conditions. This disparity could be explained by the fact that the country’s health departments provide high-quality supportive care for people living with HIV and other immunosuppressive diseases, such as diabetes and hypertension.

Furthermore, in this study, the risks of interrupting TB treatment were higher in patients who did not have a treatment supporter/contact person during the TB treatment follow-up than in patients who did have a treatment supporter. This was in line with the studies carried out in Arsi zone, Ethiopia (32) and Kenya (34). In a study from the province of Punjab, patients with pulmonary tuberculosis who obtained support from healthcare providers had a lower risk of interrupting TB treatment than those who had no support (32).

### Limitations

The present study has some limitations. A retrospective study is likely to be less accurate and reliable than a prospective cohort study design because it is subject to bias and lacks key variables. The study was limited by unrecorded outcomes and insufficient information on basic sociodemographic, behavioral, clinical, and other tuberculosis risk factors. Consequently, the study fails to include all risk factors for treatment interruption. Furthermore, there might be differences in consistently capturing data by health facilities, and those registrations with incomplete data were excluded. These might underestimate or overestimate the findings of this study.

### Conclusion and recommendations

According to this study, a high incidence rate of TB treatment interruption was documented among patients with TB in the public health facilities of Nekemte town. Being younger, male, residing in a rural area, having comorbidities, and a lack of treatment supporters during the treatment follow-up were found to be predictors of interruption of TB treatment. These findings show the importance of addressing these risk factors in TB control programs to enhance treatment adherence and reduce the incidence of treatment interruption among TB patients. In addition, health facilities should provide supportive care for patients with comorbidities and consider interventions that target middle-aged patients from rural areas that reduce treatment interruptions.

## Data Availability

The original contributions presented in the study are included in the article/Supplementary Material, further inquiries can be directed to the corresponding author.
